# Ephemeroptera (Mayflies) Assemblages and Environmental Variation along Three Streams Located in the Dry-Hot Valleys of Baima Snow Mountain, Yunnan, Southwest China

**DOI:** 10.3390/insects12090775

**Published:** 2021-08-29

**Authors:** Muhammad Farooq, Xianfu Li, Lu Tan, Davide Fornacca, Yanpeng Li, Nima Cili, Zhen Tian, Lu Yang, Xiaoling Deng, Shuoran Liu, Wen Xiao

**Affiliations:** 1 Institute of Eastern-Himalaya Biodiversity Research, Dali University, Dali 671003, China; Farooq23028@gmail.com (M.F.); lixf@eastern-himalaya.cn (X.L.); fornacca@eastern-himalaya.cn (D.F.); liyp@eastern-himalaya.cn (Y.L.); tianzhen@ihb.ac.cn (Z.T.); yl1264@dingtalk.com (L.Y.); dengxiaoling5563@dingtalk.com (X.D.); 2 Collaborative Innovation Center for Biodiversity and Conservation in the Three Parallel Rivers Region of China, Dali 671003, China; 3 State Key Laboratory of Freshwater Ecology and Biotechnology, Institute of Hydrobiology, Chinese Academy of Sciences, Wuhan 430072, China; tanlu@ihb.ac.cn; 4 Deqin Administration Bureau, Baima Snow Mountain National Nature Reserve, Diqing 674501, China; nimacili2021@163.com

**Keywords:** high mountains, freshwater biodiversity, environmental heterogeneity, dry-hot valleys

## Abstract

**Simple Summary:**

Mayflies are among the most susceptible insect groups in mountain freshwater bodies, where they are facing different environmental threats, resulting in loss of species assemblage and diversity. In this study, we described the structure of the community of mayflies in three different streams in the dry-hot valley of Baima Snow Mountain, China, and assessed the potential effect of environmental variation over this specific group of insects. The results showed clear shifts in the community structure of mayflies between the streams. From the study area, 18 taxa were identified, with *Baetis* sp. and *Baetiella marginata* being the most prevalent. Overall, the Yeri stream hosted suitable habitats for several taxa of mayflies than the other two streams, as demonstrated by the highest species richness and diversity. Moreover, there was high environmental heterogeneity between the streams, in turn influencing the species of mayflies, particularly in the Sharong stream. As expected, the results also showed that some of the environmental factors such as altitude, conductivity, total dissolved solids, water temperature, dissolved silicon, and pH explained most of the variation in species composition.

**Abstract:**

Mountain freshwater ecosystems are threatened all over the world by a range of human-induced stresses, ensuing in a rapid loss of habitats and species diversity. Many macroinvertebrates are reactive to habitat disturbance, and mayflies (Ephemeroptera) are amongst the most sensitive groups. Despite they are susceptible to environmental deviation, knowledge concerning their species richness and diversity is still unknown in remote areas. The objectives of this study were to (1) investigate the mayfly species assemblage and community composition along different mountain streams and assess potential differences, and (2) identify the environmental variation and its influence on the structure of mayfly communities within such freshwater systems. We collected biological and environmental data from 35 sites situated along elevation gradients in the Baima Snow Mountain, northwest Yunnan, China. Multivariate analyses were performed on the environmental variables and the mayfly species composition, as well as on richness and diversity indices. We found that the community composition of mayflies was different across all three watercourses. Among the 18 Ephemeroptera taxa identified, *Baetis* sp. and *Baetiella marginata* were highly dominant, accounting for over 50% of the dissimilarity of each stream. In terms of species assemblages, almost all sites in the Yeri stream hosted good-quality habitats for several mayfly species, as reflected by the highest species richness. The Benzilan stream followed, whereas the Sharong stream showed relatively low mayfly assemblage. This variation was explained by the high environmental heterogeneity between the three watercourses. In particular, the RDA model revealed that among the different environmental factors analyzed, altitude, conductivity, total dissolved solids, water temperature, dissolved silicon, and pH explained most of the variation in species composition. Moreover, the altitude alone explained 17.74% of the variation, and in-depth analysis confirmed its significant effect on diversity indices. Further research should focus on evaluating the scale of threats to this important group of insects in the mountain freshwater ecosystem, particularly the impact of human-induced disturbances such as land use/landcover alterations.

## 1. Introduction

Mountain freshwater environments feature important ecosystems hosting high rates of biodiversity and species endemism, many of which are particularly sensitive to environmental conditions. Owing to increasing climate variability and human pressure in mountainous regions, several aquatic organisms are experiencing growing stress [1]. Among them, Ephemeroptera (mayflies) communities residing in the mountain pristine water bodies are an important group worldwide for water quality monitoring and biological evaluation due to their large numbers and the ability to detect the effects of pollution [2]. Ephemeroptera is a small order of insects distributed all over the world with nearly 3500 species, 450 genera, and 42 families [3], occurring in almost all types of running and standing freshwater systems [4]. Mayflies are also the main constituent of the aquatic macroinvertebrate communities and form the main component of biomass and macroinvertebrate production in freshwater habitats [5,6]. The shape and structure of their aquatic nymphs are extremely diverse, reflecting their corresponding habitats, movement, and feeding behaviors [3]. Their life cycle includes an aquatic and a terrestrial stage; nevertheless, they spend most of their lives in the aquatic environment, only emerging as winged adults to mate and lay eggs [6]. The growth rates of eggs are typically influenced by abiotic factors [7], reflecting ecological conditions [4]. The number of instars for a specific species does not seem to be constant, but it most likely varies within certain bounds on the basis of environmental factors such as food quality and temperature [6]. In terms of ecological and environmental status, nymphs of mayfly species have piqued interest not only in the study of aquatic insect populations or benthic macroinvertebrates but also in the application of biological indicators in stream ecosystems [8,9]. Mayfly population structure, seasonal dynamics, distribution, and their narrow habitat sensitivity expand their utility value beyond being markers and surrogates of habitat change [10] to being agents for comprehensive protection planning of freshwater resources [11].

Previous research showed that the existence and distribution of mayfly communities are lowering from environmentally unpolluted to slightly disturbed habitats [12]. Environmental variability is a key factor in determining the biodiversity of freshwater bodies [13] since it allows for more diverse niche partitioning [14]. Generally, when the environmental quality deteriorates, the habitat probably becomes more homogenous, resulting in a decrease of species composition, richness, and abundance pattern [15,16,17], being attributable to the commonness of more generalist species over specialist species [13]. In fact, this process may deviate the distribution of highly sensitive species [18,19]. Many studies have revealed that water temperature, substrate type, oxygen concentration, flow rate, and nutrients are the key environmental ingredients driving macroinvertebrate communities, including mayfly species [20,21,22]. In contrast, the impacts of human activities, usually involving changes in hydrological status and feeding resources, are often more rapid and intense and have adverse consequences on macroinvertebrate communities [23]. Because of these rapid alterations of natural habitats, numerous species may be lost prior to being brought to the knowledge of science [24]. Hence, conservation-oriented research or community-level analysis linked to environmental factors is still uncommon [25,26], and understanding species diversity in mountain areas is critical for protecting biodiversity and predicting species responses to upcoming ecological shifts [27]. Moreover, knowledge of aquatic ecosystems in several remote areas is very limited. Since mayflies play such an important role in the faunistic structure, a better knowledge of the ecology and distribution of Ephemeroptera in remote mountainous freshwater systems would be of great interest for better understanding their functioning. Thus, we assembled a dataset of biological and environmental variables from three streams situated in the particular environmental conditions of a dry-hot valley of Baima Snow Mountain (BSM), Yunnan, China. We aimed to understand the environmental variations and mayfly biodiversity of the mountain water ecosystem by concentrating on the following objectives: (1) investigating the mayfly species assemblage and community composition along different mountain streams and assessing potential differences, and (2) identifying the environmental variation and its influence on the structure of mayfly communities within such freshwater systems. The findings will help in clarifying the ecological significance of these particular habitats and provide baseline information to assist natural resource and landscape managers in their protection and conservation efforts.

## 2. Materials and Methods

### 2.1. Field Sampling and Lab Analysis

**Figure 1:**
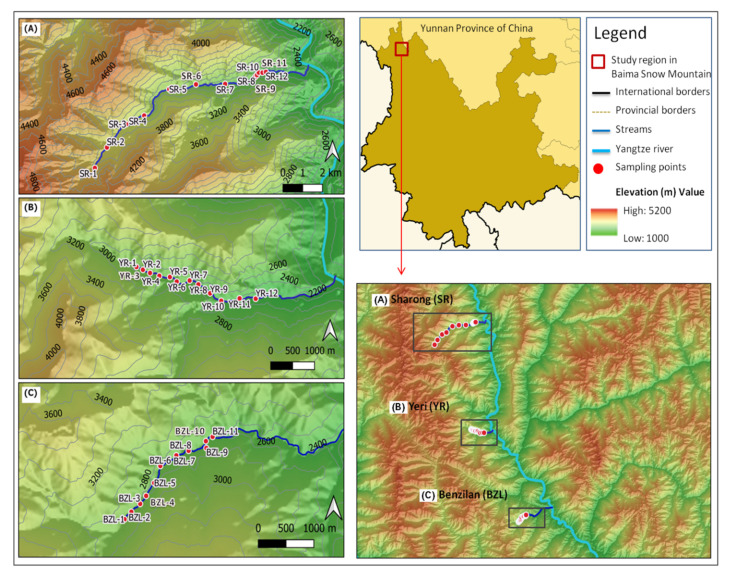
Map of the study region and the distribution of sampling sites (*n* = 35) of the three streams in Baima Snow Mountain, northwest Yunnan, China. (**A**) Sharong, (**B**) Yeri, and (**C**) Benzilan.

The study area is located in the Baima Snow Mountain (BSM) Nature Reserve (Figure 1), a central section of the Hengduan Mountains, southwest China (27°24–28°36′ N, 98°57′–99°25′ E), with elevations ranging between 2040 and 5429 m. The Hengduan Mountains are situated in the transitional zone between the Oriental and the Paleo-arctic biogeographic regions, which is one of the world’s hotspots of biodiversity [28]. This area is characterized by a cold temperate climate under the influence of the monsoon, resulting in discrete dry (November to April) and wet seasons (May to October). The weather pattern shifts with rising elevation, which makes the deep valleys hot and dry, while mountain tops are cold. Detailed information on the climate of the study area can be found in Wu et al. [29] and Wen et al. [30]. The combination of elevation and climatic gradients allows for a fairly clear discrimination of major mountain forest types by altitudinal belts.

From September to October 2017, a total of 35 sites located in the dry-hot valley environment of the Baima Snow Mountain were sampled. The sites were distributed in three tributary streams of the Yangtze River, namely, Benzilan (BZL), Sharong (SR), and Yeri (YR). These watercourses are small (maximum width of 4.5 m) and shallow, with beds composed of pebbles. Following the elevation gradient along the streams, different vegetation types covered the areas around the sample points. The sites located more upstream were relatively free from human activities, being situated far from settlements, while small-scale agricultural activities took place around the middle and downstream sites. Six sampling sites of the SR stream were located within the dry-hot valley environment with extremely low vegetation cover. Macroinvertebrate samples were collected by using a Surber net (30 × 30 cm, 500 µm, mesh size), and the materials left on the sieve were manually hand-picked and stored in 95% ethanol. At each site, five replicates were taken in order to cover a variety of habitat types. All specimens were identified and classified to genera and possibly to morphospecies level using Morse et al. [31], Dudgeon [32], and literature in the lab. For each stream site, physicochemical variables including water temperature (WT), conductivity (Cond), dissolved oxygen (DO), total dissolved solids (TDS), hydrogen ion concentration (pH), and oxidation reduction potential (ORP) were measured in situ with a multi-parameter probe (YSI Professional plus, United States) during the macroinvertebrate sampling. We also recorded the flow rate (Flow-v) using a Flow Globe FP101 (Global Water) turbidity (Turb) with a turbidity meter (SGZ–200BA, China), and the width of each stream was measured using a tape. The elevation and geographical coordinates of sampling sites were retrieved with a GPS unit (Garmin eTrex20, China). For the remaining variables, water samples were collected in pre-cleaned and polyethylene bottles and acidulated to a pH < 2 by adding sulfuric acid in the field. In the laboratory, a segmented flow analyzer (Skalar San++, the Netherlands) was used to quantify the concentration gradients of total phosphorus (TP), orthophosphate (PO_4_), total nitrogen (TN), nitrate (NO_3_), ammonia (NH_3_), salinity (Sal), dissolved silicon (Si), total organic carbon (TOC), and dissolved organic carbon (DOC).

### 2.2. Data Analysis

All data collected from 35 sites across three streams were summarized and analyzed using different statistics. According to the type of distribution, normality tested with Kolmogorv–Smirnov test, the potential environmental differences between streams were evaluated using ANOVA with post hoc Tukey HSD and nonparametric Kruskal–Wallis using post hoc Dunn test. In a second step, we identified highly correlated environmental factors in each stream separately and used the whole dataset by the means of the Spearman correlation test, setting the threshold at r ≥ 0.7 [33]. Afterward, principal component analysis (PCA) was run to analyze and plot the distribution pattern of the environmental parameters, using the Euclidean distance and z-scored standardization.

To visualize the differences in community composition of mayfly taxa in each stream, we used non-metric multidimensional scaling (NMDS) with Bray–Curtis dissimilarity index as the distance measurement. The bootstrapping PERMANOVA (adonis), a non-parametric multivariate statistical test, was used to further confirm the differences in composition (*p* < 0.05). To test for differences in dispersion between streams, we used the PERMADISP procedure using the “betadisper” function (*p* > 0.05). Moreover, the similarity percentage analysis (SIMPER) using the Bray–Curtis dissimilarity index was also performed to determine the mean contribution of each species to the mean overall dissimilarity. In particular, we were interested in identifying which taxon contributed the most to the explained variance.

Following a detrended correspondence analysis (DCA) over the mayfly community data, we selected redundancy analysis (RDA) as the appropriate ordination method (the community data were linear, the eigenvalue was below 50%, and the final length of DCA gradient value was equal to 1.661) to evaluate the correlation between environmental factors and Ephemeroptera communities. As with the PCA, the environmental variable matrix was z-score-standardized by using Euclidean distance, while the community matrix was Hellinger-transformed as required by the RDA model. The significance of the model of the RDA axes and of each of the used variables were tested by ANOVA (1000 permutations). A parallel PERMANOVA using 999 permutations and Bray–Curtis dissimilarity index was performed on environmental factors to confirm the RDA model findings.

Finally, two diversity indices, Shannon index (H) and Simpson index (1-D), were calculated for all sites of the three streams. Further, since the three streams were located at different altitudes, diversity indices were analyzed by a linear model with factor altitude in order to observe the trend of indices with this factor. All inferential and descriptive statistical analyses were conducted in the R-statistic environment [34], with the addition of the vegan package [35], while the maps were produced with QGIS 3.8 (https://www.qgis.org/, accessed on 10 January 2021).

## 3. Results

### 3.1. Environmental Variation across the Three Streams in BSM

The results of the ANOVA and Kruskal–Wallis tests showed significant differences in some of the measured environmental factors between the three streams (Table 1). Specifically, nutrients and the physical conditions contributed to the discrepancies with strong significant differences (*p* < 0.001). The factors that well-characterized these systems were Cond, TDS, pH, TN, Si, DOC, TOC, and the width of the streams, whereas the factors that did not show significant differences (*p* > 0.05) were WT, Alt, ORP, NH3, DO, TP, Flow-v, and Turb. According to Tukey’s HSD and Dunn tests for pairwise comparisons between streams, most environmental factors were usually non-significant and showed lower differences between YR and BZL streams (Table S1). The environmental factors salinity, orthophosphate, and nitrate were excluded prior to analysis due to excessive collinearity.

**Figure 2:**
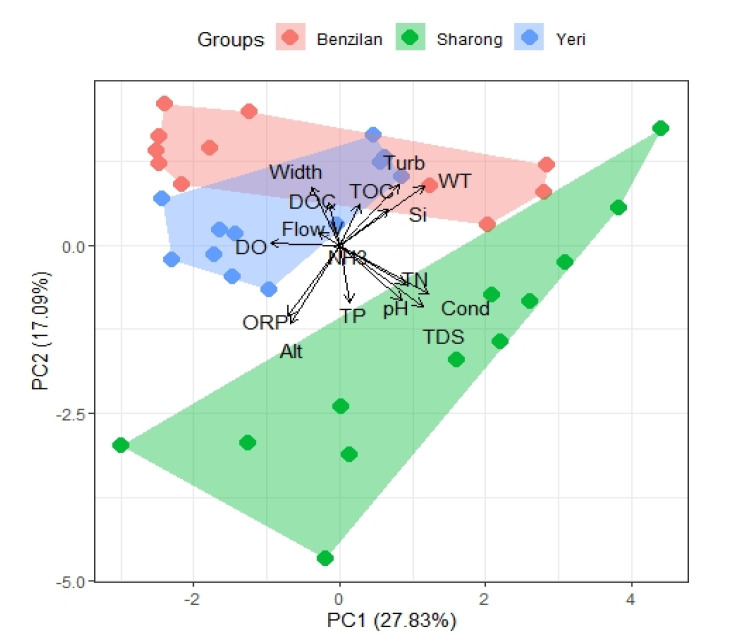
PCA biplot of physicochemical parameters based on the Euclidean distance for three streams. The variations captured by the first two PC axes are shown. Abbreviations: altitude (Alt), conductivity (Cond), total dissolved solids (TDS), water temperature (WT), dissolved oxygen (DO), hydrogen ion concentration (pH), oxidation reduction potential (ORP), total nitrogen (TN), ammonia (NH_3_), total phosphorus (TP), silicon (Si), dissolved organic carbon (DOC), chlorophyll-a (Chla), total organic carbon (TOC), width of the stream (Width), flow velocity (F-v), turbidity (Turb).

Moreover, the principal component analysis (PCA) provided further insights corroborating the differences between the three streams (Figure 2). As suggested by the pairwise difference tests, we were able to observe some degree of overlapping between the YR and BZL streams in the PCA biplot because of some environmental factors presenting statistically non-significant differences. The SR stream was clearly separated from the others along the first axis (PC1), mainly due to differences in Cond, TDS, pH, and abundant nitrogen content. Similarly, along the second axis (PC2), the streams were separated by factors such as TDS, WT, and turbidity. The first axis of the principal component explained 27.83% environmental heterogeneity, the second axis explained 17.09%, while the third axis (PC3) accounted for 12.96% of the variation. Apart from that, the length of the NH_3_ variable was very short in the PCA biplot, indicating that the first two components contained almost no information about this element. The cumulative proportion of variance for the first six principal components reached 80.36%. The third component was composed of Alt, TP, TOC, and DOC; the TP and DOC oriented opposite along the first axis; the fourth component was composed of Si; and width, the contribution of Si to the fourth component being the highest. In short, these factors greatly contributed to the first four principal components in PCA ordination and showed particularly robust differences between streams.

### 3.2. Distribution, Structure, and Composition of Mayfly Assemblages

A total of 10,658 mayfly individuals were identified in the 35 analyzed sites of the three streams, where 4570 individuals of mayflies were identified from the BZL stream, 2971 individuals from the SR stream, and 3117 from the YR stream, distributed in 8 families and 18 taxa (Table 2). The family Heptageniidae was found with the highest number of taxa and accounted for 1544 individuals and was represented by five taxa: *Iron* sp., *Epeorus* sp., *Ecdyonurus* sp., *Rhithrogena* sp1, and *Rhithrogena* sp2. The most widely distributed family was Baetidae, found in all sites of the three watercourses, recording the highest number of individuals (n = 8288) belonging to four taxa: *Baetis* sp., *Nigrobaetis (Takobia)* sp., *Baetiella marginata,* and *Baetiella spathae.* Following in abundance were the Ephemerellidae family with 491 individuals represented by three taxa: *Ephemerlla* sp., *Serratella* sp., and *Drunella* sp. The Leptophlebiidae family included one taxon: *Habrophlebiodes* sp. The families with the lowest number of individuals were Ephemeridae, which included two taxa: *Ephemera* sp. and *Ephemera wuchowensis*, and finally Ameletidae, Neoephemeridae, and Caenidae, each one represented by one taxon: *Ameletus* sp., *Potamanthellus Edmundsi,* and *Caenis* sp., respectively. More information on taxonomic abundance and richness at each stream site separately can be found in Table S2.

**Figure 3:**
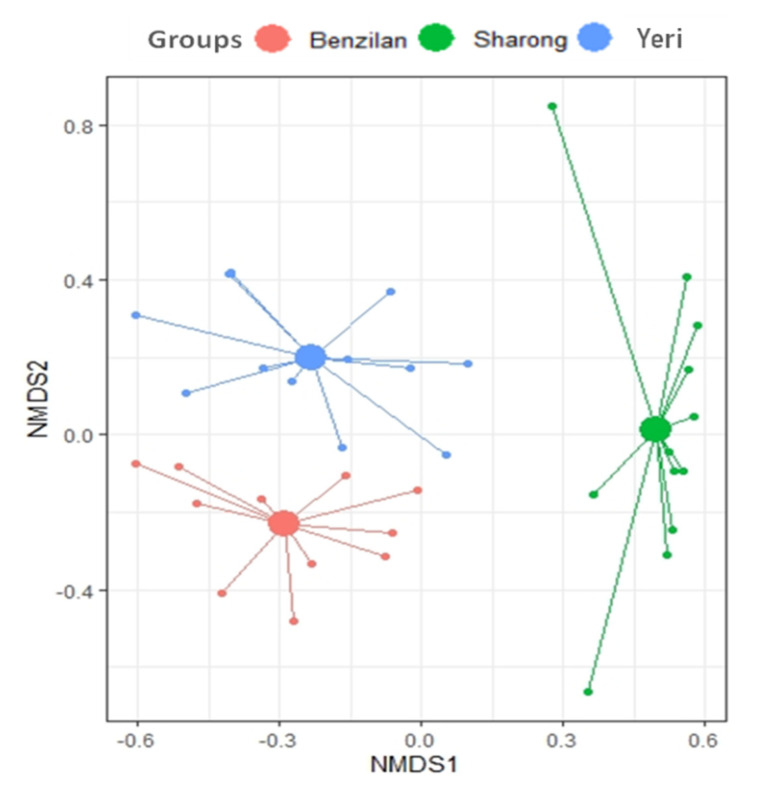
Non-metric multidimensional scaling (NMDS) plot based on Bray–Curtis dissimilarity index for the three analyzed streams, represented by colors. In this plot, it can be seen that the predicted centroid (larger-filled circles) distance displayed the similarity and dissimilarity between the three streams.

As expected, the NMDS plots showed a great distance between the species found in the three streams. The community composition of Ephemeroptera was also particularly different in the SR stream, as shown by the long distance between their centroid (Figure 3). This separation is further supported by the PERMANOVA results showing statistically significant differences in community structure across the three streams (*p* = 0.001, R^2^ = 0.4). The findings revealed a location effect on mayfly communities’ assemblage, but not a dispersion effect (e.g., the PERMANOVA output).

Additionally, the SIMPER analysis determined the contribution of each taxon (%) to the dissimilarity between streams, as shown in Table 3. The overall average dissimilarity between the streams was 53.58%. The species with a higher proportion are those who explain the group dissimilarity. In this scenario, the first two species accounted for over 50% of the dissimilarity of each stream and together with the third species accounted for more than 60%. Moreover, the taxa with a continuous high contribution to the differences across such systems are considered good discriminating taxa, In the present study, those taxa were *Baetis* sp. and *B. marginata*. However, the *Baetis* sp. was also found in environments with uncommon conditions. The cumulative percentage of the first seven species reached 90%, and the other 12 species contributed for the remaining 10%. Seven out of the overall 18 species accounted for the majority of the distinction in each stream.

### 3.3. Distribution Pattern and Relationship between Ephemeropterans and Environmental Factors

**Figure 4:**
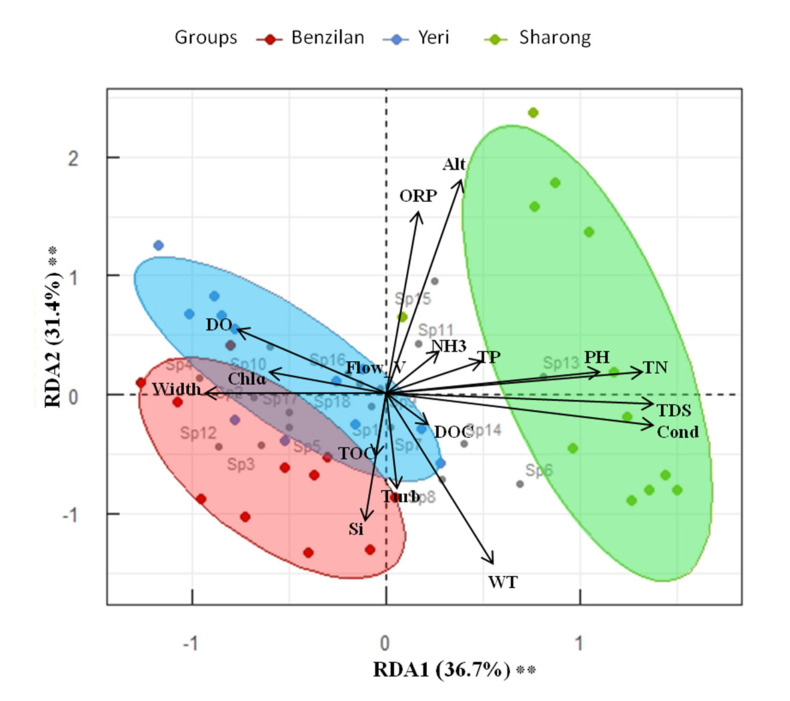
RDA model biplot based on community and environmental variable matrices. The environmental variables were used as explanatory factors (*n* = 17). The significance of the model, axes, and factors was determined by ANOVA (1000 permutations; α = 0.05). Stars next to percentages stand for the level of significance: according to the code: (*) 0.05 ≤ *p*-values < 0.01; (**) 0.01 ≤ *p*-values < 0.001; (***) *p*-values ≤ 0.001.

Generally, at the three streams scale, the distribution of taxa was significantly different according to the ANOSIM test (*p* ≤ 0.001, R^2^ = 0.6). The relationship between taxa and environmental factors is shown in Figure 4. On the basis of environmental status and community structure, the RDA biplot shows the integrated position of each stream. The degree of variation or inertia in taxa composition in the studied sites was equal to 0.05 for axis 1 and 0.04 for axis 2. The resulting RDA model with the included environmental factors explained 77.0% of the total variance, of which 68.1% was described in the first two axes (respectively, 36.7% and 31.4%; Figure 4). The constrained RDA related to the selection of an environmental factor at the time revealed that Alt, Cond, TDS, WT, Si, and pH were the indicators that explained most of the variation in species composition (Table S3). Altitude (Alt) explained the highest amount of variance (17.74%), followed by Cond (15.16%), TDS (9.09%), WT (5.08%), Si (4.63%), and pH (3.93%), while the remaining indicators explained less than 3% of the variance. PERMANOVA on the Bray–Curtis dissimilarity index confirmed the observed pattern. The first axis of the RDA was positively correlated with Alt, ORP, DO, NH3, TP, pH, and Chlɑ, while it was negatively correlated with WT, Si, Cond, TDS, TN, TOC, DOC, Width, Flow_v, and Turb of the streams. These results indicate that the first axis represents an Alt-WT gradient, these factors being strongly negatively correlated to each other. It also seems that as WT increased, DO decreased, which is an important component for the survival of aquatic organisms.

**Figure 5:**
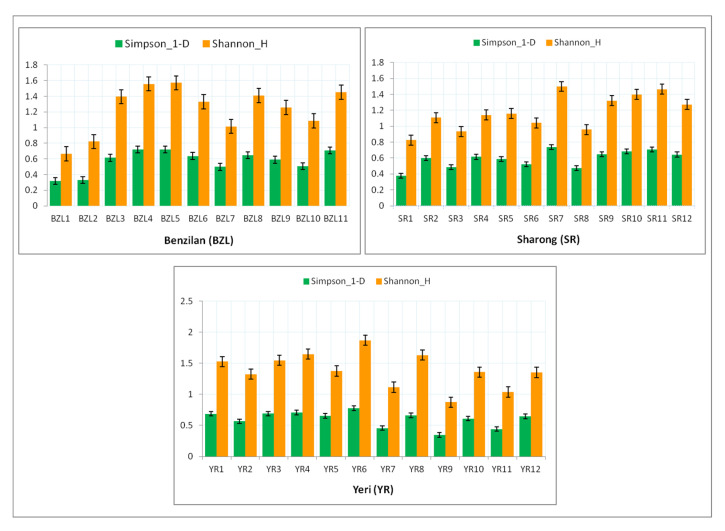
Diversity indices of mayfly taxa at various sites of the three streams. The error bars signify the standard error of average values.

In addition, Figure 5 shows the two diversity indices (Simpson-D_1 and Shannon_H) for each stream. Among the BZL sampling sites, the value of both indices was found to be higher at the head of the stream than downstream (particularly BZL4 and BZL5). In the YR stream, the values of such diversity indices were similarly found to be higher at sites near the mountain’s summit (YR4 and YR6). However, in the SR stream, the maximum diversity values were recorded downstream (SR11 and SR7). Comparatively, the YR stream was slightly more diverse and richer than the other two streams, but in contrast with low abundance in comparison with BZL. In general, most sites of the YR stream served as a suitable habitat for several species of mayflies, followed by BZL, with the exception of the SR stream, which showed a relatively low mayfly diversity.

**Figure 6:**
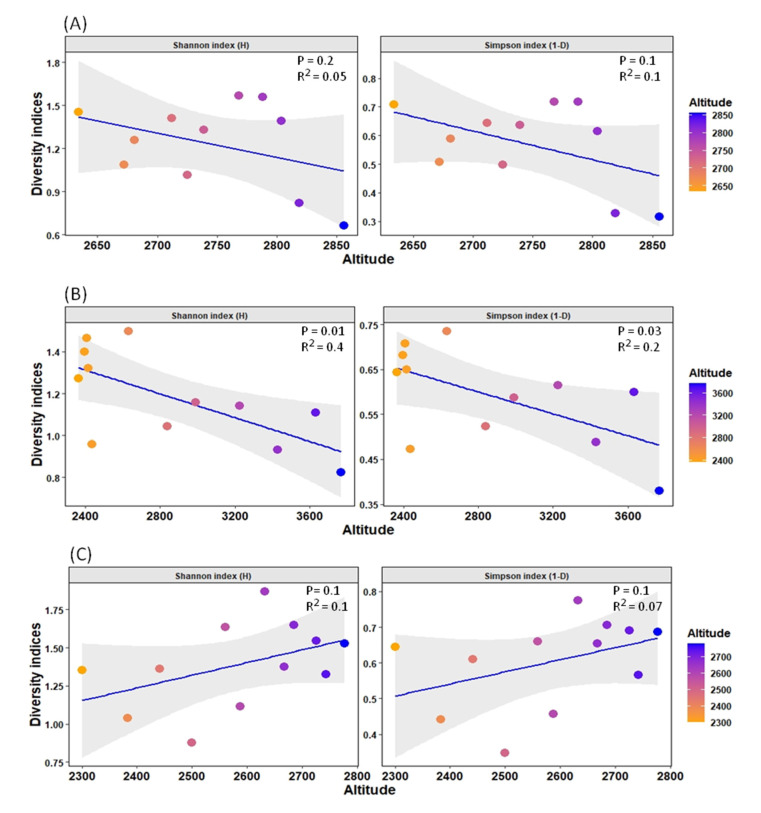
Relationship between diversity indices (Shannon index (H) and Simpson index (1-D)) and altitude gradient for each stream. The grid colors represent low and high altitudes in the graph. (**A**) BZL stream, (**B**) SR stream, and (**C**) YR stream.

Furthermore, due to the high percentage of variance explained by altitude in the distribution pattern (17.74% in the RDA model), an additional analysis of diversity indices was undertaken to observe the diversity patterns of such streams with altitude. It was observed that diversity in the SR stream was significantly negatively correlated with the factor altitude, while diversity in the BZL stream was not significantly negatively correlated. The YR stream, on the other hand, showed a non-significant positive correlation (Figure 6).

## 4. Discussion

Research in ecology has identified several environmental factors such as water temperature, dissolved oxygen, nutrient resources, and human disturbance as having direct influences on community structure and biodiversity [36,37]. Understanding the effect of these factors on aquatic organisms is very important, particularly with regard to mayflies because of their high susceptibility to slight changes in stream conditions. The three freshwater ecosystems located in the dry-hot valley of Baima Snow Mountain investigated in this study showed significant environmental variation, which was confirmed by different tests on individual variables and a PCA ordination. Additional insights into the variation within each stream were revealed; in particular, one of the streams, the Sharong (SR), had a wider range of environmental variation than the other two and showed a clear dynamic status of environmental gradients. Six among the 12 sampling sites in this stream were located within the dry-hot valley environment, where the stream flows between two mountains flanks covered by bare soil. Because of the destruction of the original vegetation cover, the natural ecology and habitat of this area have changed considerably [38], in turn influencing the structure of different mayfly species. Moreover, all sites of the SR stream also had higher pH and altitude values than the other two streams. According to Svitok [39] and Vilenica et al. [10], pH has significant effects on mayfly species composition, such as the regulation of main physiological functions. For example, the ion trade with water and respiration cannot occur at a relatively narrow range of pH (<6.5 and >8.5) [40]. The pH values observed at all sites in BZL (except site 11) were under the prescribed range (6.0 to 8.5) for standard aquatic life [41], while in the YR stream, except for some sites, the pH values were also within the specified range. The high pH recorded in the SR stream indicated an alkaline environment, which is most likely to cause a relatively low mayfly species assemblage [42,43]. As expected, the RDA model revealed that Alt, Cond, TDS, WT, Si, and pH were the indicators explaining most of the variation in species composition. Similar indicators have been identified to influence mayfly species assemblage in other studies [44,45,46]. Among them, water temperature is the most important environmental factor affecting mayfly communities’ structure. This is due to the fact that temperature is closely related to the embryonic development, nymphal growth, metabolism, emergence, and survival rate of many species [47]. Warm-adapted insects, such as many mayflies, grow faster and are active at higher temperatures rather than during their winter resting (quiescence) [48]. Similarly, conductivity (Cond) and total dissolved solids (TDS) are related parameters. For example, an experiment performed by Olson and Hawkins [46] found that the taxa that performing better in the moderate conductivity treatment had the maximum conductivity optima, showing that low TDS concentrations may limit the distribution of taxa adapted to higher TDS concentrations. The authors inferred that the taxa that can tolerate low conductivity conditions will probably experience higher stress than taxa that prefer higher conductivity habitats, as stream total dissolved solids (TDS) rises in response to the undergoing climate and land-use change. Pond [49] and Pond et al. [50] found that increased conductivity was harmful to several macroinvertebrate groups, particularly Ephemeroptera species, with a fair amount of mayflies dropping dramatically in streams with conductivity surpassing 175 µs/cm^–1^. The effect of water temperature, Cond, and TDS on macroinvertebrates, including Ephemeroptera species, has been confirmed by many researchers [46,51].

Among other variables, altitude is one of the major factors affecting macroinvertebrate distribution patterns, including mayfly species in mountain freshwater bodies [52,53]. In our study, we found that the diversity indices value of the YR stream is significantly higher than that of the other two streams, indicating that almost all sites of this stream served as suitable habitat for several species of mayflies, followed by BZL. However, due to its high environmental heterogeneity, the SR stream showed relatively low mayfly species assemblage, which might not be favorable to many species of mayflies, and most of the sites were inhabited by widespread and generalist species. Moreover, the diversity indices were negatively impacted by altitude in the SR and BZL streams (Figure 6). These findings are aligned with other studies indicating that with increasing altitude, diversity decreases [54], ascribed to limiting physicochemical factors, such as harsh climatic conditions or decreased resource availability at high altitudes. For example, the negative relation of stream macroinvertebrates diversity (including mayflies) with altitude indicates the dissolved oxygen concentration according to Jacobsen [55], because most of the macroinvertebrates, including the mayflies, rely directly on the oxygen concentration since they usually possess gills or other underwater breathing systems [56]. In general, Ephemeroptera is particularly more diverse in temperate and tropical environments [3], and their diversity declines toward the poles owing to less suitable habitats [54]. Conversely, in the YR stream, the diversity indices were non significantly positively correlated with altitude. In general, many researchers have examined the variance in diversity as a function of altitude and drawn various conclusions. For example, in Costa Rica streams, Pringle and Ramirez [57] found higher diversity at higher altitudes than at lower altitudes. Similarly, Tate and Heiny [58] found a positive association between richness and altitude in a catchment of the Colorado River. They ascribed this relationship to the higher rate of human activities at lower altitudes, which caused a reduction in species diversity. These findings signal to further deeply investigate the diversity patterns of mayfly species along altitudinal gradients to better understand these variations in such remote freshwater systems.

Meanwhile, the community composition of mayflies across three streams was significantly different according to NMDS and PERMANOVA tests. Moroever, the NMDS biplot showed more variation in community structuring between SR-BZL and SR-YR, which could have been due to the SR stream’s high environmental variability. Comparatively, the highest species richness and diversity were recorded in the YR stream, followed by BZL stream, whereas the lower richness was observed in the SR stream (Table S2). The higher diversity and taxonomic richness of the YR stream can be attributed to high water quality and good habitat. In addition, the SIMPER analysis determined *Baetis* sp. and *B. marginata* as the mayfly species contributing the most to inter- and intra-stream dissimilarity, which means that they can be considered as good discriminating species (Table 3). However, average dissimilarity was rather low for most mayfly species. This may have been due to the community structure being very similar across the different environments of such streams. Throughout the study, *Baetis* sp. alone represented 39.2% of the differences in each stream. Moreover, the presence of this taxon in a variety of environmental conditions indicates a certain resistance to the effect of gradients (generalist mayfly). In other studies, the genera *Baetis* and *Caenis* have shown resistance to change [59,60]. Nevertheless, the *Caenis* sp. is viewed as a rare species, and other engrossing species, such as *Potamanthellus edmundsi*, *Ephemerlla* sp., *Ameletus* sp., *Ephemera* sp., and *Ephemera wuchowensis*, were among the rarest in our study. These species might have relatively low ecological plasticity and thus did not typically occur at most sampling sites.

There is a clear lack of research on Ephemeroptera species in northwest Yunnan, and more taxonomic investigation is suggested to better understand the function of each species in different environments. The increased taxonomic resolution makes biological and ecological studies more meaningful. At present, published information on the mayfly fauna of northwest Yunnan is still poor, and community-based research requiring precise levels of identification is difficult to implement. The present study in three streams of Baima Snow Mountain represents a building step for the establishment of a basic understanding of the Ephemeroptera fauna in the region. An undefined number of taxa still needs to be discovered, and extended research is needed to understand the effects of anthropogenic pressure on freshwater ecosystems, such as changes in the terrestrial environment interacting with the aquatic habitats and the residing species.

## 5. Conclusions

Freshwater ecosystems are an important area for macroinvertebrate biodiversity, including mayfly species. This study investigated mayfly species assemblage and environmental heterogeneity in three streams located in a dry-hot valley of Baima Snow Mountain freshwater streams, revealing a marked shift in composition and structure within various sites along an elevational gradient. In such areas, there is high environmental heterogeneity and several niches for different assemblages and species composition of mayflies. These findings inform on the significant peculiarities and potential uniqueness of every alpine stream. Mountain freshwater systems are highly vulnerable and require a comprehensive conservation strategy, from the lowlands to the top of the mountains, and from the local to the watershed scale. This works also provides a strong foundation that would allow us to well comprehend the overall structure and function of mountains’ complex ecosystems. Bearing this in mind, further research encourages the assessment of the level of threats to these important groups of insects, as one of the indicators of mountains’ ecological status.

## Figures and Tables

**Table 1:** Basic statistics of physicochemical parameters and results of the ANOVA and nonparametric Kruskal–Wallis for the three streams. The superscripts “*” and “***’’ indicate a statistically significant effect at *p* < 0.05 and *p* < 0.001, respectively, while “ns” stands for not significant (*p* > 0.05).

Environmental Factors	Benzilan			Sharong			Yeri		
	Max	Min	Mean ± Std	Max	Min	Mean ± Std	Max	Min	Mean ± Std
Alt (m) ^ns^	2856	2634	2743.27 ± 75.8	3769	2364	2878.5 ± 520.95	2778	2300	2583.75 ± 151.21
Cond (µs/cm) ***	305.0	84.1	159.064 ± 99.6	287.3	172	254.958 ± 36.83	170.0	139.4	151.392 ± 10.21
TDS ***	247.6	75.40	134.963 ± 80.3	250.9	167.7	219.158 ± 23.66	142.35	126.10	133.466 ± 5.07
WT (°C) ^ns^	14.6	10.2	12.082 ± 1.44	15.6	7.5	12.000 ± 2.87	13.3	10.1	11.200 ± 1.25
DO (mg/L) ^ns^	8.39	6.98	7.530 ± 0.40	7.78	6.94	7.398 ± 0.31	8.20	7.27	7.665 ± 0.29
pH ***	8.86	7.91	8.437 ± 0.27	8.96	8.62	8.863 ± 0.08	8.91	8.11	8.678 ± 0.21
ORP ^ns^	132.2	75.3	97.727 ± 21.11	138.1	62.1	102.058 ± 27.87	115.2	36.6	85.642 ± 25.08
TN (mg/L) ***	0.15	0.09	0.118 ± 0.02	0.39	0.16	0.260 ± 0.06	0.22	0.18	0.194 ± 0.01
NH_3_ (mg/L) ^ns^	0.019	0.004	0.008 ± 0.00	0.018	0.005	0.009 ± 0.00	0.019	0.03	0.008 ± 0.00
TP (mg/L) ^ns^	5.006	3.754	4.256 ± 0.55	4.335	2.719	3.548 ± 0.57	3.512	3.033	3.173 ± 0.15
Si (mg/L) ***	0.034	0.016	0.022 ± 00.00	0.118	0.014	0.030 ± 0.02	0.026	0.013	0.015 ± 0.00
DOC (mg/L) *	1.58	0.48	1.103 ± 0.35	2.02	0.71	0.975 ± 0.35	1.08	0.58	0.780 ± 0.14
Chla ^ns^	0.078	0.031	0.493 ± 0.01	0.189	0.000	0.031 ± 0.05	1.183	0.009	0.124 ± 0.33
TOC (mg/L) ***	3.060	0.943	1.373 ± 0.57	2.31	0.54	0.875 ± 0.52	0.765	0.485	0.582 ± 0.07
Width (m) ***	4.5	1.8	2.982 ± 0.84	2	2	2.00 ± 0.00	5.0	2.5	3.708 ± 0.75
Flow-v (m/s) ^ns^	1.00	0.32	0.574 ± 0.18	0.80	0.44	0.558 ± 0.10	0.74	0.24	0.471 ± 0.15
Turb (NTU) ^ns^	2.8	0.5	1.573 ± 0.66	5.3	0.0	1.200 ± 1.70	1.8	0.0	0.983 ± 0.67

Note: The abbreviations of different factors are: altitude (Alt), conductivity (Cond), total dissolved solids (TDS), water temperature (WT), dissolved oxygen (DO), hydrogen ions concentration (pH), oxidation reduction potential (ORP), total nitrogen (TN), ammonia (NH_3_), total phosphorus (TP), silicon (Si), dissolved organic carbon (DOC), chlorophyll-a (Chla), total organic carbon (TOC), width of the stream (Width), flow velocity (F-v), turbidity (Turb).

**Table 2:** Ephemeroptera taxa and their overall abundance and distribution in the three analyzed streams: Benzilan (BZL), Sharong (SR), and Yeri (YR).

Families	Taxa	Benzilan	Sharong	Yeri	Total
Neoephemeridae	*Potamanthellus edmundsi*	1	0	1	2
Ephemerellidae	*Ephemerlla* sp.	2	0	16	45
Ephemerellidae	*Serratella* sp.	36	0	11	47
Ephemerellidae	*Drunella* sp.	190	0	209	399
Baetidae	*Baetis* sp.	2821	1098	1806	5725
Baetidae	*Nigrobaetis (Takobia)* sp.	330	282	174	786
Baetidae	*Baetiella marginata*	701	548	232	1481
Baetidae	*Baetiella spathae*	174	43	75	292
Siphlonuridae	*Ameletus* sp.	3	1	0	4
Leptophlebiidae	*Habrophlebiodes* sp	7	1	306	314
Caenidae	*Caenis* sp.	0	1	0	1
Heptageniidae	*Iron* sp.	206	14	74	294
Heptageniidae	*Epeorus* sp.	12	116	59	187
Heptageniidae	*Ecdyonurus* sp.	21	46	31	98
Heptageniidae	*Rhithrogena* sp1.	30	820	109	959
Heptageniidae	*Rhithrogena* sp2.	1	1	4	6
Ephemeridae	*Ephemera* sp.	8	0	9	17
Ephemeridae	*Ephemera wuchowensis*	0	0	1	1
Total		4570	2971	3117	10,658

**Table 3:** The analysis of similarity percentage (SIMPER) for mayfly taxa in the three analyzed streams. The SIMPER analysis determines the percentage contribution of each species to the Bray–Curtis dissimilarity index.

Taxon	Average Dissimilarity	Percentage Contribution	Cumulative Percentage
*Baetis* sp.	21.01	39.21	39.21
*Baetiella marginata*	8.552	15.96	55.17
*Rhithrogena* sp1	7.106	13.26	68.43
*Nigrobaetis (Takobia)* sp.	3.762	7.021	75.45
*Habrophlebiodes* sp.	3.121	5.825	81.28
*Drunella* sp.	2.988	5.577	86.85
*Iron* sp.	2.156	4.025	90.88
*Baetiella spathae*	1.873	3.495	94.37
*Epeorus* sp.	1.198	2.236	96.61
*Ecdyonurus* sp.	0.707	1.321	97.93
*Ephemerlla* sp.	0.420	0.784	98.71
*Serratella* sp.	0.375	0.700	99.42
*Ephemera* sp.	0.147	0.274	99.69
*Rhithrogena* sp2	0.055	0.103	99.79
*Ameletus* sp.	0.032	0.061	99.92
*Potamanthellus edmundsi*	0.025	0.048	99.97
*Ephemera wuchowensis*	0.009	0.016	99.98
*Caenis* sp.	0.008	0.015	100

Note: The first column identifies the species explained by that row; the second and third columns show the average dissimilarity and percentage contribution elucidated in species in descending order, respectively; and the fourth column tallies the cumulative percentage for these species represented in the table.

## Data Availability

Data can be provided upon request to the authors by email contact (Farooq23028@gmail.com).

## Supplementary Materials

The following are available online at https://www.mdpi.com/article/10.3390/insects12090775/s1, Table S1: Results of the post-hoc TukeyHSD and Dunn tests for inter-group differences for each environmental component. Table S2: The species abundance, species richness, and species mean values for each study site of the three analyzed streams. The bold numbers for each stream point to the highest number of species abundance and species richness respectively. Table S3: The results of the parallel PERMANOVA and associated ANOVA in the RDA model for each environmental indicator.
