# Supplementary material for: Ephemeroptera (Mayflies) Assemblages and Environmental Variation along Three Streams Located in the Dry-Hot Valleys of Baima Snow Mountain, Yunnan, Southwest China
Source: Insects. 2021 Aug 29;12(9):775. doi: 10.3390/insects12090775 (PMC8466216; doi:10.3390/insects12090775)
Supplement: Supplementary file 1 [file insects-12-00775-s001.zip › insects-1314667-supplementary/Table S1.pdf]

Table S1. Results of the post-hoc TukeyHSD and Dunn tests for inter-group differences for each environmental component.

Environmental Factors	Streams	Diff	Lwr	Upr	*p* Value
Total dissolved solid	SR-BZL	84.19470	35.88243	132.50697	< **0.001**
	YR-BZL	-1.49697	-49.80924	46.81530	0.996
	YR-SR	-85.69167	-132.94200	-38.44133	< **0.001**
Altitude (Alt)	SR-BZL	135.2273	-193.8440	464.29856	0.576
	YR-BZL	-159.5227	-488.5940	169.54856	0.466
	YR-SR	-294.7500	-616.5881	27.08807	0.077
Conductivity (Cond)	SR-BZL	95.89470	34.30936	157.48003	< **0.001**
	YR-BZL	-7.67197	-69.25731	53.91337	0.949
	YR-SR	-103.56667	-163.79831	-43.33502	< **0.001**
Water temperature (WT)	SR-BZL	-0.081818	-2.142594	1.978957	0.994
	YR-BZL	-0.881818	-2.942594	1.178957	0.550
	YR-SR	-0.800000	-2.815478	1.215478	0.597
Dissolved oxygen (DO)	SR-BZL	-0.1325758	-0.4764341	0.2112826	0.614
	YR-BZL	0.1349242	0.1349242	0.4787826	0.604
	YR-SR	0.2675000	-0.0688001	0.6038001	0.140
Hydrogen ion concentration (pH)	SR-BZL	0.4260606	0.2173285	0.6347928	**0.001**
	YR-BZL	0.2410606	0.03232853	0.44979268	**0.02**
	YR-SR	-0.1850000	-0.3891439	0.0191439	0.081
Oxidation reduction potential (ORP)	SR-BZL	4.331061	-21.26565	29.927772	0.909
	YR-BZL	-12.085606	-37.68232	13.511105	0.485
	YR-SR	-16.416667	-41.45074	8.617411	0.255
Total nitrogen (TN)	SR-BZL	0.14265152	0.09862609	0.18667694	< **0.001**

	SR-BZL	0.07598485	0.07598485	0.12001027	< **0.001**
	YR-SR	-0.0666666	-0.10972438	-0.0236089	**0.001**
Ammonia (NH3)	SR-BZL	0.00102272	0.00102272	0.00526538	0.825
	YR-BZL	-0.0002272	-0.0044699	0.00401538	0.990
	YR-SR	0.00289939	-0.00289939	0.00289939	0.741
Total phosphorus (TP)	SR-BZL	0.00873484	-0.00859294	0.02606263	**0.02**
	YR-BZL	-0.0065984	-0.02392627	-0.0239262	0.622
	YR-SR	-0.0153333	-0.03228024	0.00161357	0.082
Dissolved oxygen concentration (DOC)	SR-BZL	-0.1286364	-0.4327461	0.17547339	0.557
	YR-BZL	-0.3228030	-0.6269128	-0.6269128	**0.03**
	YR-SR	-0.1941667	-0.4915919	0.10325854	0.258
Chla	SR-BZL	-0.0177976	-0.2208717	0.1852765	0.974
	YR-BZL	0.07525006	-0.1278241	0.2783242	0.637
	YR-SR	0.09304767	-0.1055627	0.2916581	0.490
Total organic carbon (TOC)	SR-BZL	-0.4987273	-0.9566955	-0.0407590	**0.03**
	YR-BZL	-0.7908106	-1.2487789	-0.3328423	< **0.001**
	YR-SR	-0.2920833	-0.7399851	0.15581847	0.259
Turbidity (Turb)	SR-BZL	-0.3727273	-1.541133	0.7956781	0.715
	YR-BZL	-0.5893939	-1.757799	0.5790114	0.439
	YR-SR	-0.2166667	-1.359390	0.9260563	0.887
Silicon (Si)	SR-BZL	-0.7078485	-1.1885486	-0.2271483	**0.002**
	YR-BZL	-1.0822652	-1.5629653	-0.6015650	< **0.001**
	YR-SR	-0.3744167	-0.8445507	0.09571735	0.139
Width	SR-BZL	-0.9818182	-1.64424929	-0.3193871	**<0.01**
	YR-BZL	0.7265152	0.06408404	1.3889463	**0.02**
	YR-SR	1.7083333	1.06046293	2.3562037	< **0.001**

Flow velocity (Flow-v)	SR-BZL	-0.0162121	-0.1727890	0.14036472	0.964
	YR-BZL	-0.1028787	-0.2594556	0.05369805	0.254
	YR-SR	-0.0866666	-0.2398018	0.06646850	0.357
