# Supplementary material for: Ephemeroptera (Mayflies) Assemblages and Environmental Variation along Three Streams Located in the Dry-Hot Valleys of Baima Snow Mountain, Yunnan, Southwest China
Source: Insects. 2021 Aug 29;12(9):775. doi: 10.3390/insects12090775 (PMC8466216; doi:10.3390/insects12090775)
Supplement: Supplementary file 1 [file insects-12-00775-s001.zip › insects-1314667-supplementary/Table S2.pdf]

**Table S2.** The species abundance, species richness, and species mean values for each study site of the three analyzed streams. The bold numbers for each stream point to the highest number of species abundance and species richness respectively.

| | Benzilan (BZL) | | | Sharong (SR) | | | Yeri (YR) | | |
|---|---|---|---|---|---|---|---|---|---|
| **Sites No** | **Species Abundance** | **Species Richness** | **Species Mean** | **Species Abundance** | **Species Richness** | **Species Mean** | **Species Abundance** | **Species Richness** | **Species mean** |
| **1** | 602 | 9 | 31.68 | 285 | 5 | 15.00 | 216 | 8 | 11.37 |
| **2** | 525 | 11 | 27.63 | 362 | **8** | 19.05 | 354 | 11 | 18.63 |
| **3** | 449 | 12 | 23.63 | 555 | 7 | 29.21 | **361** | **14** | 19.00 |
| **4** | 261 | 10 | 13.74 | 190 | 6 | 10.00 | 243 | 13 | 12.79 |
| **5** | 287 | **13** | 15.11 | 318 | 6 | 16.74 | 85 | 9 | 4.47 |
| **6** | **623** | 11 | 32.79 | **564** | 7 | 29.68 | 340 | 12 | 17.89 |
| **7** | 451 | 9 | 23.74 | 141 | 6 | 7.42 | 177 | 11 | 9.32 |
| **8** | 300 | 10 | 15.79 | 170 | 7 | 8.95 | 339 | 13 | 17.84 |
| **9** | 188 | 9 | 9.89 | 121 | 7 | 6.37 | 311 | 11 | 16.37 |
| **10** | 429 | 11 | 22.58 | 117 | 7 | 6.16 | 186 | 10 | 9.79 |
| **11** | 456 | 10 | 24.00 | 73 | 7 | 3.84 | 235 | 11 | 12.37 |
| **12** | | | | 77 | 7 | 4.05 | 271 | 11 | 14.26 |

**Note:** The sites No for each stream are represented, indicating each stream with a specific site name (BZL1, BZL2, BZL3, BZL4 BZL5, BZL6, BZL7, BZL8, BZL9, BZL10, BZL11; SR1, SR2, SR3, SR4, SR5, SR6, SR7, SR8, SR9, SR10, SR11, SR12; YR1 YR2, YR3, YR4, YR5, YR6, YR7, YR8, YR9, YR10, YR11, YR12) as shown in the Figure 1.
