# Supplementary material for: Ephemeroptera (Mayflies) Assemblages and Environmental Variation along Three Streams Located in the Dry-Hot Valleys of Baima Snow Mountain, Yunnan, Southwest China
Source: Insects. 2021 Aug 29;12(9):775. doi: 10.3390/insects12090775 (PMC8466216; doi:10.3390/insects12090775)
Supplement: Supplementary file 1 [file insects-12-00775-s001.zip › insects-1314667-supplementary/Table S3.pdf]

Table S3. Results of the parallel PERMANOVA and associated ANOVA in the RDA model for each environmental indicator.

Factors \ Statistical test	PERMANOVA		ANOVA	
	P-value	R^2	*P-value*	*Variance*
**Alt**	0.001	0.411	< 0.001	17.74 %
**Cond**	0.001	0.217	< 0.001	15.16 %
**TDS**	0.01	0.206	< 0.001	9.09 %
**WT**	0.001	0.223	0.001	5.08 %
**DO**	0.03	0.097	> 0.05	1.52 %
**PH**	0.03	0.191	0.02	3.93 %
**ORP**	0.001	0.109	0.08	2.68 %
**Si**	0.001	0.141	0.005	4.63 %
**TN**	0.001	0.226	> 0.05	2.32 %
**NH3**	> 0.05	0.015	> 0.05	1.83 %
**TP**	> 0.05	0.046	> 0.05	1.75 %
**DOC**	> 0.05	0.011	> 0.05	2.01 %
**Chla**	> 0.05	0.034	> 0.05	1.05 %
**TOC**	> 0.05	0.059	> 0.05	1.64 %
**Width**	> 0.05	0.108	> 0.05	2.11 %
**Flow_V**	> 0.05	0.003	> 0.05	2.47 %
**Turb**	0.001	0.102	> 0.05	1.98 %
**Residual**				22.92 %
